# Leishmaniasis: pathogenesis, host immunity, and the evolving landscape of immunotherapy and immunochemotherapy

**DOI:** 10.3389/fmicb.2026.1861933

**Published:** 2026-06-24

**Authors:** Godspower N. Okeke, Ugochukwu K. Oduwe, Giorgi Kenkebashvili, Shefa Tabassum, Alexandre F. Marques

**Affiliations:** Department of Biological Sciences, University of Southern Mississippi, Hattiesburg, MS, United States

**Keywords:** cutaneous leishmaniasis, drug resistance, host immunity, immunochemotherapy, immunotherapy, Leishmaniasis, miltefosine, mucosal vaccination

## Abstract

Leishmaniasis is a group of neglected tropical diseases caused by protozoan parasites of the genus *Leishmania*, transmitted by the bite of infected female phlebotomine sandflies. Manifesting as cutaneous (CL), mucocutaneous (MCL), and visceral (VL) forms, it remains a major global health burden, affecting millions in tropical and subtropical regions, with an estimated 30,000 new VL cases annually and over 1 billion people at risk. The disease is defined not only by the parasite but by the host immune response: protective Th1-mediated cellular immunity, characterized by IFN-γ, TNF-α, IL-12, and nitric oxide production, is essential for parasite clearance, while Th2 skewing, IL-10 dominance, and immune anergy permit progressive disease. Current chemotherapy relies on a limited and problematic drug arsenal. Pentavalent antimonials face widespread resistance, particularly in the Indian subcontinent. Amphotericin B achieves high cure rates but demands hospitalization and causes significant nephrotoxicity. Miltefosine, the only oral agent available, achieves 94–97% initial cure rates in Indian VL (Bihar region) but is complicated by teratogenicity, gastrointestinal toxicity, 10–20% relapse rates, and emerging resistance. No licensed vaccine exists for human leishmaniasis. This narrative review synthesizes current understanding of *Leishmania* pathogenesis and host immunity, evaluates the treatment landscape across drug classes, and critically examines immunotherapy and immunochemotherapy strategies, including cytokine-based approaches, TLR agonists, therapeutic vaccines, and novel platforms such as *α*-Gal epitope-targeted virus-like particles (VLPs), as strategies to restore protective immunity and address the limitations of chemotherapy alone. We also assess mucosal vaccination as an underexplored delivery route with documented advantages for Th1 polarization and tissue-resident memory induction. Together, these approaches point toward a future of combination immunochemotherapy capable of achieving durable cure, preventing relapse and post-kala-azar dermal leishmaniasis (PKDL), and overcoming drug resistance across clinical forms of leishmaniasis.

## 1 Introduction

Leishmaniasis encompasses a spectrum of diseases caused by more than 20 pathogenic *Leishmania* species, transmitted through the bite of infected female phlebotomine sandflies (*Lutzomyia* spp. in the New World; *Phlebotomus* spp. in the Old World). The disease is endemic in over 90 countries across Africa, Asia, the Americas, and the Mediterranean basin, disproportionately affecting impoverished communities with limited access to healthcare. The World Health Organization (WHO) estimates approximately 700,000 to one million new cases annually across all forms, with visceral leishmaniasis responsible for roughly 30,000 deaths per year and representing the second most deadly parasitic disease after malaria (World Health Organization, 2024; Alvar et al., 2012; Murray et al., 2005).

Clinical manifestations span from self-healing cutaneous lesions to life-threatening systemic infection. Cutaneous leishmaniasis (CL), the most common form, presents with skin ulcers that can lead to disfiguring scars (Scott and Novais, 2016). Mucocutaneous leishmaniasis (MCL) involves destructive lesions of the nasopharyngeal mucosa. Visceral leishmaniasis (VL), caused by *L. donovani* and *L. infantum/chagasi*, is characterized by fever, splenomegaly, hepatomegaly, weight loss, and pancytopenia; if untreated, VL is almost universally fatal (Kaye and Scott, 2011). Post-kala-azar dermal leishmaniasis (PKDL), a dermal sequela following apparent cure of VL, further complicates control efforts by serving as a reservoir for transmission (World Health Organization, 2024; Zijlstra, 2016).

The central determinant of disease outcome is the host immune response. A protective Th1 response, characterized by IFN-γ, TNF-α, IL-12, and activation of inducible nitric oxide synthase (iNOS) in macrophages, drives parasite killing and resolution of infection. Conversely, Th2 cytokines (IL-4, IL-13) and regulatory IL-10 suppress macrophage activation and promote disease progression and chronicity. This immunological dichotomy has been extensively studied in murine models and validated in human clinical disease, and forms the scientific basis for all immunotherapy and immunochemotherapy strategies reviewed here (Alexander and Brombacher, 2012; Faleiro et al., 2014; Sacks and Noben-Trauth, 2002).

Current treatment remains anchored in a chemotherapy arsenal with significant limitations: toxicity, emerging drug resistance, high cost, parenteral administration requirements, and a universal failure to restore durable protective immunity (Croft et al., 2006; Ponte-Sucre et al., 2017). No licensed human vaccine exists (Noazin et al., 2008). Against this background, immunotherapy and immunochemotherapy—combining antileishmanial drugs with immunomodulators, vaccines, or novel biological platforms—represent the most promising frontier for achieving curative outcomes and long-term protection (Roatt et al., 2014; Yadagiri et al., 2023). This review provides a comprehensive and critical synthesis of Leishmania pathogenesis, host immunity, the current drug landscape, and advances in immunotherapy and immunochemotherapy across clinical forms, with attention to emerging and underexplored strategies.

## 2 Literature search and selection

This narrative review was conducted by searching PubMed, Scopus, Web of Science, and Google Scholar for English-language articles published between January 2000 and March 2025. Search terms included combinations of “leishmaniasis,” “*Leishmania donovani*,” “*Leishmania infantum*,” “*Leishmania braziliensis*,” “visceral leishmaniasis,” “cutaneous leishmaniasis,” “mucocutaneous leishmaniasis,” “host immunity,” “Th1 polarization,” “*α*-Gal epitope,” “virus-like particles,” “intranasal vaccination,” “mucosal immunity,” “miltefosine,” “immunochemotherapy,” “TLR agonists,” “drug resistance,” “PKDL,” and related terms. Inclusion criteria focused on peer-reviewed original research articles, systematic reviews, and clinical or preclinical studies relevant to *Leishmania* pathogenesis, immune evasion, drug efficacy and resistance, vaccine development, or immunotherapy across all clinical forms. Conference abstracts, non-English articles, and studies with no direct relevance to treatment or immunity in Leishmania infection were excluded. Additional sources were identified through citation chaining and expert knowledge of the field.

Narrative synthesis was employed rather than systematic meta-analysis because the retrieved evidence spans fundamentally heterogeneous designs — *in vitro* assays, murine and Syrian hamster models, canine vaccine trials, and human Phase I–III studies — rendering standardized data extraction and quantitative pooling inappropriate; the review’s aim is thematic and mechanistic integration across these domains rather than estimation of summary effects. Reporting follows SWiM guidance (Campbell et al., 2020) where applicable. As with any narrative synthesis, conclusions are subject to author judgment in source selection and interpretation, a limitation addressed in Section 8.

## 3 Pathogenesis and immune evasion

### 3.1 Parasite biology and life cycle

Leishmania parasites are obligate intracellular *kinetoplastid protozoa* that exist in two major life stages: the flagellated promastigote form in the sandfly vector, and the non-flagellated intracellular amastigote form within mammalian host macrophages and other phagocytic cells (de Morais et al., 2015; Kaye and Scott, 2011). Following inoculation by a sandfly bite, metacyclic promastigotes encounter complement and are phagocytosed by dermal macrophages, dendritic cells, and neutrophils at the bite site. Within the phagolysosome, promastigotes differentiate into amastigotes, which are uniquely adapted to survive and replicate in the acidic, hydrolytically active environment of the mature phagolysosome (de Morais et al., 2015; Olivier et al., 2005).

The clinical outcome of infection is determined by the infecting species, parasite burden, host genetic background, and, critically, the balance of the host immune response (Sacks and Noben-Trauth, 2002; Murray et al., 2005). *L. donovani and L. infantum* disseminate from the skin to visceral organs (spleen, liver, bone marrow), causing VL, including bone marrow as an important additional site of parasite establishment and maintenance (Carneiro and Peters, 2021; World Health Organization, 2024). *L. braziliensis* is primarily associated with CL and MCL. *L. amazonensis* and *L. major* cause predominantly CL with variable severity depending on host immune status (Scott and Novais, 2016; World Health Organization, 2024).

The immunopathological consequences of infection differ substantially across disease forms and should not be conflated. In CL caused by *L. major*, a self-limiting Th1 response ultimately controls infection in immunocompetent hosts, with lesion resolution and development of long-lasting protective immunity. In MCL caused by *L. braziliensis*, an exaggerated Th1/Th17 inflammatory response drives destructive immunopathology in the nasopharyngeal mucosa — parasite burden is characteristically low but tissue damage severe, illustrating that robust Th1 immunity is not uniformly protective. In VL caused by *L. donovani* and L. infantum, progressive Th1 dysfunction driven primarily by IL-10-mediated immunoregulation and T-cell exhaustion — rather than Th2 dominance per se — permits systemic dissemination and fatal organ failure if untreated. These divergent trajectories are summarized in Table 1 and inform disease-specific immunotherapy design throughout this review.

**Table 1:** Divergent immunopathological trajectories across clinical forms of leishmaniasis.

Disease form (causative species)	Dominant immune response	Key cytokines/Mediators	Parasite burden	Clinical outcome	Immunotherapy implication
Cutaneous Leishmaniasis (CL) *L. major, L. tropica*	Protective, self-limiting Th1 response	IFN-γ↑, TNF-α↑, IL-12↑, nitric oxide↑	Moderate; controlled by adaptive immunity	Ulcerative skin lesions; spontaneous resolution; development of long-lasting protective immunity	Enhance Th1 polarization; therapeutic vaccines may accelerate resolution and prevent recurrence
Mucocutaneous Leishmaniasis (MCL) *L. braziliensis*	Exaggerated Th1/Th17 immunopathology; low IL-10 regulation	IFN-γ↑↑, TNF-α↑↑, IL-17↑; IL-10↓	Low parasite burden despite severe pathology	Destructive nasopharyngeal/mucosal tissue damage; disfigurement; illustrates Th1 is not uniformly protective	Immune modulation to dampen excess inflammation while preserving parasite control; anti-TNF adjunctive strategies
Visceral Leishmaniasis (VL/Kala-azar) *L. donovani, L. infantum*	Progressive Th1 dysfunction; IL-10-mediated immunoregulation and T-cell exhaustion	IL-10↑↑, IL-12↓, IFN-γ↓; PD-1↑, CTLA-4↑	High; systemic dissemination to spleen, liver, bone marrow	Fatal if untreated; 10–20% develop PKDL post-treatment; major transmission reservoir	Restore Th1 via TLR agonists, IL-12/IFN-γ supplementation, or prophylactic VLP vaccination; immunochemotherapy to prevent relapse and PKDL

CL, cutaneous leishmaniasis; MCL, mucocutaneous leishmaniasis; VL, visceral leishmaniasis; PKDL, post-kala-azar dermal leishmaniasis; IFN-γ, interferon-gamma; TNF-α, tumor necrosis factor-alpha; IL, interleukin; PD-1, programmed death-1; CTLA-4, cytotoxic T-lymphocyte antigen-4; TLR, Toll-like receptor.

### 3.2 Immune evasion strategies

Leishmania has evolved a sophisticated repertoire of strategies to subvert host immunity and establish persistent infection within macrophages (Olivier et al., 2005; Tibúrcio et al., 2019). Key mechanisms include: (1) inhibition of complement-mediated lysis through surface molecules such as lipophosphoglycan (LPG) and gp63, which also facilitate receptor-mediated entry without triggering pro-inflammatory signaling; (2) modulation of phagosome maturation, including inhibition of phagosome-lysosome fusion and disruption of the vacuolar ATPase to prevent full acidification; (3) alteration of TLR2/TLR4 signaling to suppress pro-inflammatory cytokine cascades; (4) induction of IL-10 production, which drives macrophage deactivation and T-cell anergy; and (5) interference with MHC class II antigen presentation to prevent effective Th1 priming (Rodrigues et al., 2016; Tibúrcio et al., 2019; Olivier et al., 2005).

**Figure 1:**
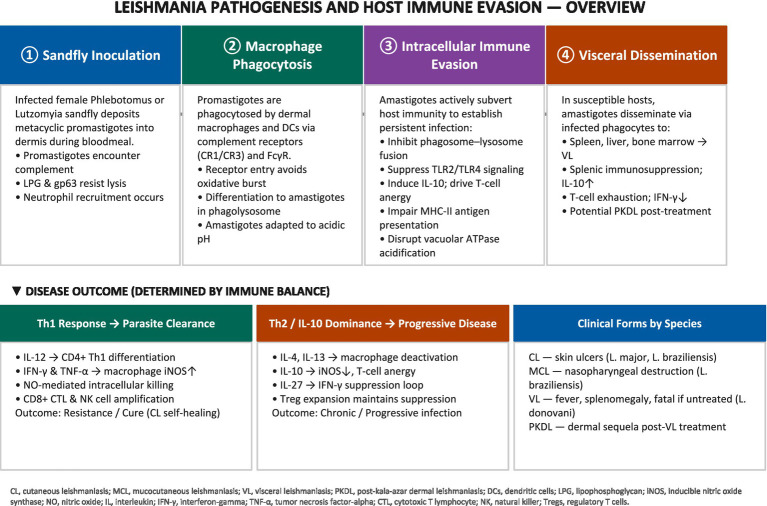
Leishmania pathogenesis and host immune evasion. Following sandfly inoculation, metacyclic promastigotes are phagocytosed by dermal macrophages (①②), differentiate into amastigotes within the phagolysosome (③), and actively evade host immunity through suppression of TLR signaling, inhibition of phagosome–lysosome fusion, and induction of IL-10-mediated T-cell anergy (④). In susceptible hosts, visceral dissemination leads to spleen, liver, and bone marrow infection. Disease outcome is determined by the host immune balance: Th1-dominant responses (IFN-γ, iNOS/NO) drive parasite clearance; Th2/IL-10-dominant responses promote chronic progressive disease. CL, cutaneous leishmaniasis; MCL, mucocutaneous leishmaniasis; VL, visceral leishmaniasis; PKDL, post-kala-azar dermal leishmaniasis; IL, interleukin; IFN-γ, interferon-gamma; iNOS, inducible nitric oxide synthase; NO, nitric oxide.

In visceral leishmaniasis (VL), progressive infection is characterized by splenic and hepatic immunosuppression, elevated IL-10 and IL-27, loss of T-cell proliferative responses, and eventual immune exhaustion (Faleiro et al., 2014; Dayakar et al., 2019; Engwerda et al., 2004). Recovery from VL is associated with restoration of IFN-γ responses and macrophage activation, underscoring the primacy of the cellular immune arm in parasite control (Faleiro et al., 2014; Dayakar et al., 2019) (see Figure 1).

## 4 Host immunity in leishmaniasis

### 4.1 Protective Th1 response

Resistance to Leishmania infection is canonically associated with a CD4 + Th1 response producing IFN-γ and TNF-α, which activate macrophages to upregulate iNOS and generate nitric oxide (NO) for intracellular parasite killing (Alexander and Brombacher, 2012; Sacks and Noben-Trauth, 2002). IL-12 from dendritic cells and macrophages is the master polarizing cytokine driving Th1 differentiation; IL-18 amplifies IFN-γ production synergistically with IL-12. Th1 responses also drive production of IgG2a antibodies in mice (IgG1 in humans), which can opsonize parasites and contribute to ADCC-mediated killing (Alexander and Brombacher, 2012; Engwerda et al., 2004).

CD8 + cytotoxic T lymphocytes (CTLs) play a critical secondary role, particularly in visceral disease, where they mediate killing of infected macrophages via perforin/granzyme pathways and amplify IFN-γ signals. Natural killer (NK) cells provide early IFN-γ during the innate phase before adaptive responses are established. Regulatory T cells (Tregs) and IL-10-producing type 1 regulatory T cells (Tr1 cells) contribute to immunopathology by suppressing effector responses, particularly during chronic infection (Faleiro et al., 2014; Murray, 1997).

### 4.2 Disease-promoting Th2 and regulatory responses

Disease susceptibility is associated with Th2 cytokine dominance (IL-4, IL-13) and immunosuppressive IL-10 production, which collectively deactivate macrophages, downregulate iNOS, and impair T-cell proliferation (Sacks and Noben-Trauth, 2002; Rodrigues et al., 2016). IL-10 is produced by multiple cell types in leishmaniasis—including macrophages, Tregs, and even exhausted CD4 + effector T cells—and is the central mediator of immune anergy in progressive VL. IL-27, produced by dendritic cells and macrophages during chronic infection, further suppresses IL-12 and IFN-γ and promotes IL-10, creating a self-reinforcing suppressive loop (Rodrigues et al., 2016; Dayakar et al., 2019).

The failure to sustain Th1 responses explains both the clinical progression of untreated VL and the relapse observed after chemotherapy alone: even when parasites are reduced to undetectable levels by drug treatment, incomplete immune restoration allows residual parasites to re-expand upon treatment cessation (Rijal et al., 2013; Dorlo et al., 2012). This immunological rationale underpins immunochemotherapy approaches that pair parasite-killing drugs with immune restoration strategies (Roatt et al., 2014; Yadagiri et al., 2023).

### 4.3 Beyond the Th1/Th2 paradigm: emerging immunological mechanisms

Immune exhaustion and checkpoint regulation. PD-1, LAG-3, and TIM-3 upregulation on CD4 + and CD8 + T cells drives functional exhaustion in chronic VL, profoundly impairing effector responses. Critically, IL-10-mediated immunoregulation — produced by macrophages, Tregs, Tr1 cells, and exhausted CD4 + T cells — is now recognized as more central to VL disease progression than canonical Th2 polarization alone (Rodrigues et al., 2016; Faleiro et al., 2014). Checkpoint blockade (anti-PD-1/PD-L1) has been proposed as an adjunct immunotherapy strategy to reinvigorate exhausted T cells in VL, though safety in the context of HIV-VL coinfection requires careful evaluation (see Section 4.4).

Macrophage metabolic reprogramming. Leishmania infection drives a shift in macrophage metabolism toward aerobic glycolysis that suppresses oxidative phosphorylation and NO-mediated intracellular killing. Pro-inflammatory Th1 cytokine signaling (IFN-γ, TNF-α) reverses this metabolic shift, restoring microbicidal capacity. Metabolic immunotherapy approaches — including AMPK-activating agents such as metformin and modulators of the itaconate pathway — are being explored as potential adjuncts to conventional antileishmanial therapy.

Trained innate immunity. Transient exposure of monocytes and macrophages to Leishmania antigens or adjuvants (including BCG) can induce durable epigenetic reprogramming — histone acetylation and methylation changes at promoters of pro-inflammatory genes — that confers heightened innate responsiveness to subsequent challenges, termed trained immunity. This mechanism may contribute to the non-specific protection observed with BCG-adjuvanted killed whole-parasite vaccines and represents a targetable pathway for Leishmania prophylaxis independent of adaptive T-cell priming.

Follicular helper T cells (Tfh) and humoral dysfunction. Tfh cells drive germinal center reactions and shape the isotype and avidity maturation of anti-Leishmania antibody responses. In active VL, expansion of atypical B-cell populations and dysfunctional germinal center activity contribute to impaired long-term humoral immunity (Faleiro et al., 2014), with implications for vaccine durability and for antibody-dependent immunotherapy platforms such as alpha-Gal VLPs, whose efficacy depends on high-avidity IgG responses.

Th17 cells and IL-17. IL-17 contributes to neutrophil recruitment and early innate control in *L. major* CL models, where it plays a broadly protective accessory role. In VL, however, elevated IL-17 is associated with immunopathological tissue damage rather than parasite clearance (Dayakar et al., 2019), illustrating that the same cytokine can have opposing functional roles across disease forms — an important species-specific nuance for immunotherapy design.

The Th1/Th2 binary framework, while useful as a conceptual scaffold for immunotherapy rationale, is now recognized as an oversimplification of Leishmania immunology, particularly in human VL. Several additional immunological mechanisms shape disease outcome and must be incorporated into a complete model of host–parasite interaction.

### 4.4 HIV–visceral Leishmaniasis coinfection: an extreme test of host immunity

HAART-associated immune reconstitution inflammatory syndrome (IRIS) following antiretroviral therapy initiation is a recognized complication in HIV-VL coinfection and can unmask or exacerbate VL-associated immunopathology. Immunotherapy approaches that reinvigorate T-cell responses — particularly checkpoint blockade strategies — carry a theoretical risk of worsening IRIS and must be approached with caution in coinfected patients. The narrow immunological window created by effective HAART-mediated CD4 + recovery may represent the most viable opportunity for therapeutic immunotherapy in this population.

Relapse rates in HIV-VL coinfection are substantially higher than in HIV-negative VL, estimated at 25–60% after standard antileishmanial treatment, because the host cannot sustain the immune reconstitution necessary to control residual parasites after drug treatment ends. PKDL occurs at higher rates in HIV-VL-coinfected patients despite apparent VL cure, reflecting persistent immunosuppression. Liposomal amphotericin B is the treatment of choice in coinfection, with combination drug regimens under ongoing evaluation.

HIV-VL coinfection represents one of the most clinically important contexts of immune failure in leishmaniasis, with HIV coinfection rates reaching 30–40% of VL cases in some East African cohorts, and has direct implications for immunotherapy design. The two infections create a mutually reinforcing cycle of immunosuppression: Leishmania drives IL-10-mediated macrophage deactivation and T-cell exhaustion, while HIV directly depletes CD4 + T cells, removing the Th1 priming capacity required to contain the parasite. Below a CD4 + T-cell count of approximately 200 cells/mm^3^, effective Th1 priming — and consequently protective vaccination — is not achievable.

### 4.5 Mucosal immunity in Leishmaniasis

An underappreciated dimension of anti-Leishmania immunity is mucosal immunity, particularly secretory IgA (SIgA) and tissue-resident memory T cells (TRM). Intranasal vaccination studies have demonstrated that mucosal delivery of Leishmania antigens induces Th1 responses that are comparable to or exceed those of parenteral routes under optimal formulation conditions, with IFN-γ production both locally at mucosal surfaces and systemically, as well as reduced Th2 cytokines (Pratti et al., 2016; de Matos Guedes et al., 2014; Helou et al., 2021).

**Figure 2:**
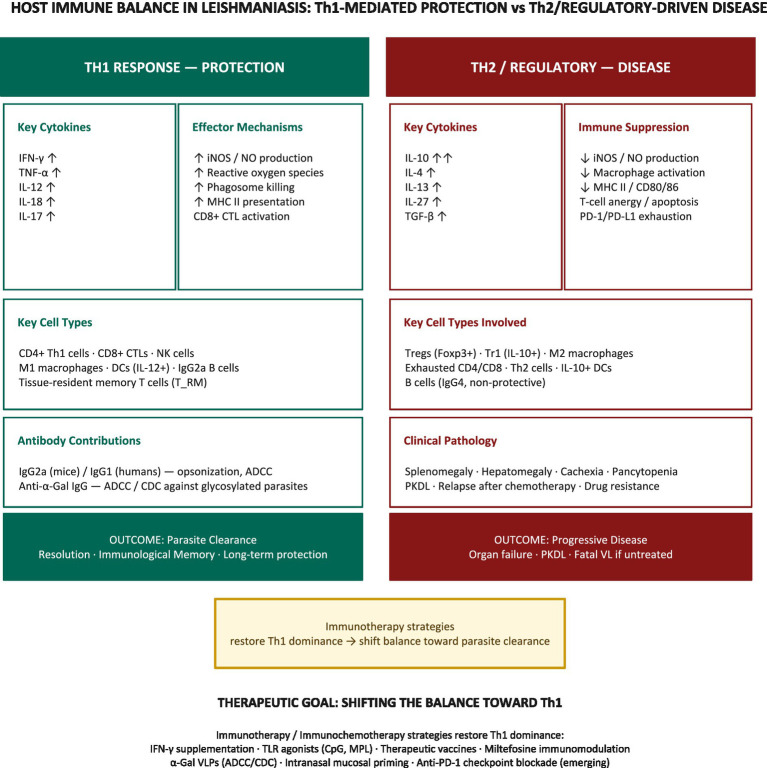
Host immune balance in leishmaniasis: Th1-mediated protection versus Th2/regulatory-driven progressive disease. Th1 dominance activates macrophage iNOS/NO production and drives parasite clearance; Th2/regulatory dominance suppresses macrophage microbicidal function and promotes progressive disease. Immunotherapy strategies restore Th1 dominance to achieve durable parasite control. IFN-γ, interferon-gamma; TNF-α, tumor necrosis factor-alpha; IL, interleukin; iNOS, inducible nitric oxide synthase; NO, nitric oxide; Tregs, regulatory T cells.

Intranasal delivery activates antigen-presenting cells in mucosa-associated lymphoid tissues (MALT), driving retinaldehyde dehydrogenase (RALDH) activity in dendritic cells, promoting SIgA production and T cells with mucosal homing profiles (α4β7+, CCR9+) (Holmgren and Czerkinsky, 2005; Lycke, 2012; Farazuddin et al., 2019). Tissue-resident memory T cells provide long-term surveillance at sites of parasite entry and persistence. The role of SIgA in mucosal protection has been extensively characterized across infectious disease models (Corthésy, 2013). These mucosal immune mechanisms are not efficiently induced by parenteral vaccination, suggesting that the route of immunization is a significant determinant of vaccine efficacy in leishmaniasis (Lavelle and Ward, 2021; Sharma et al., 2020; Lo et al., 2012) (see Figure 2).

Intranasal vaccination carries specific risks that must qualify its application. Antigen delivery to the nasal mucosa without adequate adjuvant can induce tolerance rather than immunity, because nasal-associated lymphoid tissue (NALT) maintains a homeostatic bias toward regulatory T-cell induction for soluble antigens (Lavelle and Ward, 2021). An ISCOM-adjuvanted intranasal influenza vaccine was associated with Bell’s palsy in Switzerland, prompting its withdrawal and reassessment of mucosal adjuvant safety standards (Mutsch et al., 2004). Common mucosal adjuvants — CpG ODN, cholera toxin B, ISCOM systems — are temperature-sensitive and pH-labile, a practical constraint in cold-chain-limited endemic settings. Efficacy advantages shown in inbred murine models have not been reproduced in outbred or non-human primate studies, and no intranasal Leishmania vaccine candidate has entered clinical trials. Intranasal delivery is conditionally advantageous, not categorically superior, and its application depends on antigen, adjuvant, and formulation selection.

## 5 Current treatment landscape

### 5.1 Pentavalent Antimonials

Pentavalent antimonials (meglumine antimoniate and sodium stibogluconate) were the first-line treatment for all forms of leishmaniasis for over seven decades. Their mechanism involves intracellular conversion to trivalent antimony (Sb^3+^), which inhibits key metabolic enzymes including trypanothione reductase and type I topoisomerase in Leishmania amastigotes. Standard treatment requires 20–30 days of intramuscular or intravenous injection, with significant adverse effects including cardiotoxicity (QT prolongation, arrhythmia), pancreatitis, and hepatic injury (Chakravarty and Sundar, 2010; Didwania et al., 2017; Haldar et al., 2011).

The most critical limitation of pentavalent antimonials is widespread drug resistance, particularly in the Indian subcontinent. In Bihar, India—historically the epicenter of VL—resistance rates exceeding 60% have effectively removed antimonials from clinical use, necessitating the adoption of alternative agents (Bhandari et al., 2012; Croft et al., 2006). Resistance mechanisms include downregulation of aquaglyceroporin channels that mediate drug uptake, overexpression of efflux pumps (MRPA), increased trypanothione biosynthesis, and reduced Sb^5+^-to-Sb^3+^ conversion (Bhusal et al., 2025; Hefnawy et al., 2017; Ponte-Sucre et al., 2017).

### 5.2 Amphotericin B

Amphotericin B deoxycholate achieves cure rates exceeding 95% in VL and is the recommended second-line agent in antimonial-resistant areas. It acts by binding ergosterol in the Leishmania membrane, forming pores that disrupt osmotic integrity and cause parasite death. Despite its efficacy, conventional amphotericin B carries substantial nephrotoxic risk, requires prolonged hospitalization and IV infusion, and is not practical in resource-limited endemic settings (Berman, 2015; Sundar et al., 2004; Sundar and Chakravarty, 2013).

Liposomal amphotericin B (AmBisome) significantly reduces toxicity by encapsulating the drug in lipid bilayers, enabling preferential delivery to reticuloendothelial tissues where Leishmania resides. Single-dose and short-course regimens have achieved >95% cure in clinical trials (Sundar et al., 2004; Savoia, 2015). However, the cost of $150–200 per treatment course limits access in endemic regions, and WHO prequalification and procurement programs have partially but incompletely addressed this barrier (Savoia, 2015; Singh et al., 2012). Nanoparticle-based amphotericin B formulations, including pH-sensitive nanostructured lipid carriers, represent active areas of formulation research aimed at reducing cost and enabling topical or oral delivery (Zhang et al., 2025).

### 5.3 Miltefosine

Miltefosine is the only orally administered antileishmanial agent currently approved for clinical use and the most significant advance in VL treatment since the introduction of antimonials. Originally developed as an anticancer agent, miltefosine disrupts Leishmania via multiple mechanisms: perturbation of membrane phospholipid composition and ergosterol biosynthesis, mitochondrial dysfunction, inhibition of GPI anchor biosynthesis, disruption of calcium homeostasis, and induction of programmed cell death-like apoptosis in the parasite (Dorlo et al., 2012; Shivahare et al., 2014; Sundar and Olliaro, 2007).

The landmark Phase III clinical trial by Jha et al. established miltefosine as an effective oral agent with 94% cure rates in Indian VL (Jha et al., 1999). Standard 28-day regimens achieve 94–97% initial cure rates in Indian VL (Bihar region) (Sundar et al., 2006; Bhattacharya et al., 2004). However, miltefosine faces critical limitations: teratogenicity mandates contraception in women of reproductive age; gastrointestinal adverse effects (nausea, vomiting, diarrhea) occur in 38–60% of patients; and critically, relapse rates of 10–20% are observed, particularly in East Africa. Relapse is linked to reduced drug uptake via mutations or downregulation of the LdMT/LdRos3 transporter complex, sub-therapeutic drug exposure due to pharmacokinetic variability and patient-specific factors, and—crucially—failure to restore durable protective immunity following parasite clearance (Rijal et al., 2013; Dorlo et al., 2012; Ponte-Sucre et al., 2017).

Beyond direct leishmanicidal activity, miltefosine exerts important immunomodulatory effects. It promotes Th1 polarization, upregulating IFN-γ, IL-12, and TNF-α while suppressing IL-4 and IL-10. It enhances macrophage iNOS/NO production, upregulates MHC class II and costimulatory molecules (CD80/CD86), and restores T-cell lymphoproliferation in anergic VL patients. These immunomodulatory properties are mechanistically important for immunochemotherapy combinations, as miltefosine can amplify vaccine-induced immunity while simultaneously reducing parasite burden (Shivahare et al., 2014; Dorlo et al., 2012).

The 10–20% relapse rates observed with miltefosine reflect at least three mechanistically distinct failure modes that must not be conflated. Parasitological resistance involves genetically encoded changes in the parasite that reduce drug susceptibility — most commonly downregulation or loss-of-function of the LdMT/LdRos3 inward transporter complex, upregulation of ABC efflux activity, and altered membrane lipid composition reducing drug incorporation — confirmed in clinical isolates from relapsing patients in Nepal and East Africa (Rijal et al., 2013; Bhusal et al., 2025). Pharmacokinetic failure refers to sub-therapeutic systemic drug exposure in the absence of intrinsic parasite resistance, arising from interindividual variability in drug clearance, body-weight-dependent underdosing (particularly in underweight pediatric patients), incomplete adherence over the 28-day regimen, or pharmacogenomic variation in drug metabolism (Dorlo et al., 2012). Immunological failure describes the inability to restore durable protective Th1 immunity following successful initial parasite reduction, permitting residual parasites to re-expand in a persistently immunosuppressive host environment after treatment cessation — a mechanism that persists even when pharmacokinetic exposure is adequate and the parasite retains full *in vitro* drug sensitivity. Immunochemotherapy is principally designed to address immunological failure, and may reduce the parasite burden threshold at which pharmacokinetic failure becomes clinically significant; it does not directly address parasitological resistance.

### 5.4 Paromomycin

Paromomycin (aminosidine), an aminoglycoside antibiotic, inhibits protein synthesis in Leishmania by binding to the 16S rRNA subunit of the small ribosomal unit, disrupting translational fidelity. It is administered parenterally (intramuscularly) for 21 days and achieves 60–95% efficacy with significant regional variability (Hailu et al., 2010; Barrett and Croft, 2012). Primary adverse effects include ototoxicity and nephrotoxicity, particularly at higher doses. Paromomycin is relatively low-cost and has been incorporated into combination regimens with amphotericin B and miltefosine to shorten treatment duration and reduce resistance risk (Singh and Sundar, 2023; Hailu et al., 2010; see Table 2).

**Table 2:** Clinical characteristics and limitations of current visceral leishmaniasis therapies.

Drug	Efficacy	Route/Duration	Key limitations
Pentavalent antimonials	35–65% (resistant areas)	IV/IM, 20–30 days	Widespread resistance (Bihar, India); cardiotoxicity, pancreatitis, hepatotoxicity
Amphotericin B deoxycholate	>95%	IV, hospitalized	Severe nephrotoxicity; requires hospitalization; not field-deployable
Liposomal AmB (AmBisome)	>95%	IV, short course	Cost $150–200/course; limits access in endemic regions
Miltefosine	94–97% initial	Oral, 28 days	Teratogenicity; GI toxicity (38–60%); 10–20% relapse; PKDL risk
Paromomycin	60–95% (variable)	IM, 21 days	Ototoxicity; regional efficacy variability; parenteral only

Data adapted from Didwania et al. (2017), Barrett and Croft (2012), Dorlo et al. (2012), and World Health Organization (2024). PKDL, post-kala-azar dermal leishmaniasis; IM, intramuscular; IV, intravenous; GI, gastrointestinal (World Health Organization, 2024; Dorlo et al., 2012; Didwania et al., 2017; Bhattacharya et al., 2004).

### 5.5 Drug resistance mechanisms

Resistance to antileishmanial drugs has emerged as one of the most pressing challenges in leishmaniasis control (Ponte-Sucre et al., 2017; Croft et al., 2006). Leishmania’s remarkable genomic plasticity—including chromosomal aneuploidy, gene amplification via extrachromosomal amplicons, and large genomic rearrangements—enables rapid phenotypic adaptation to drug pressure without the fixed mutations typically associated with bacterial antibiotic resistance (Bhusal et al., 2025; Garcia-Hernandez et al., 2012; Hefnawy et al., 2017).

For antimonials, resistance is primarily mediated by reduced drug activation, increased Sb^3+^ efflux via MRPA, elevated trypanothione levels sequestering Sb^3+^, and reduced AQP1/AQP3 aquaglyceroporin expression limiting drug influx (Bhusal et al., 2025; Bhandari et al., 2012; Hefnawy et al., 2017). For miltefosine, resistance involves downregulation or inactivating mutations of the LdMT/LdRos3 inward transporter complex, altered membrane lipid composition reducing drug incorporation, and enhanced efflux via ABC transporters. Cross-resistance between miltefosine and amphotericin B has been observed, and the molecular determinants are incompletely understood, raising concern about the durability of combination regimens (Bhusal et al., 2025; Bhandari et al., 2012; Ponte-Sucre et al., 2017).

**Figure 3:**
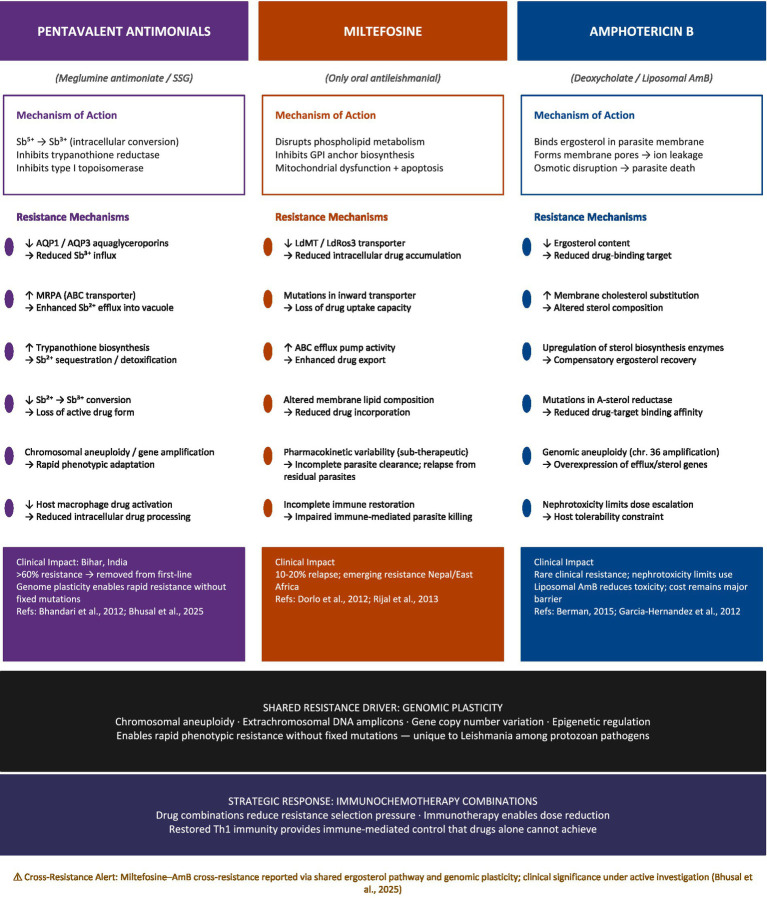
Mechanisms of drug resistance in Leishmania. Resistance pathways for pentavalent antimonials (reduced aquaglyceroporin expression, MRPA efflux, elevated trypanothione), miltefosine (LdMT/LdRos3 transporter downregulation, ABC efflux), and amphotericin B are shown with clinical impacts. Shared genomic plasticity—including chromosomal aneuploidy and gene amplification—underlies cross-drug resistance. Immunochemotherapy combinations reduce drug selection pressure and enable dose reduction. MRPA, multidrug resistance protein A; AQP, aquaglyceroporin; ABC, ATP-binding cassette.

Epigenetic mechanisms—including histone modifications, DNA methylation, and non-coding RNAs—have been implicated in regulating resistance-associated gene expression (Bhusal et al., 2025; Hefnawy et al., 2017), though their functional contributions require further validation through gene knockout approaches. The rapid acquisition of resistance phenotypes, combined with the limited drug pipeline, underscores the urgency of immunotherapy and immunochemotherapy strategies that reduce drug dose requirements and restore immune-mediated control to achieve durable parasite clearance (Roatt et al., 2014; Yadagiri et al., 2023; Chatelain and Ioset, 2011) (see Figure 3).

## 6 Immunotherapy approaches

### 6.1 Cytokine-based immunotherapy

The earliest immunotherapy approaches for leishmaniasis leveraged IFN-γ, the master activating cytokine for macrophage-mediated parasite killing. Systemic IFN-γ administration combined with pentavalent antimonials demonstrated cure rates of 75–100% in murine VL models and 88–100% in clinical trials in India and East Africa, compared to antimonials alone (Dayakar et al., 2019; Sundar et al., 2004; Murray, 1997). However, systemic IFN-γ administration is limited by flu-like adverse effects, high cost, and the logistical challenges of parenteral cytokine delivery in resource-limited settings (Dayakar et al., 2019).

Combinations of IFN-γ with IL-17A and with colony-stimulating factors (GM-CSF) have demonstrated enhanced parasite clearance in experimental models, with GM-CSF improving macrophage activation and antigen presentation capacity. TNF-α-amplifying strategies and IL-12 supplementation have shown promise in restoring Th1 responses in anergic VL patients (Murray, 1997; Masic et al., 2023). Adjunct therapy with rIFN-γ and rIL-17A alongside sub-optimal amphotericin B effectively *controlled L. donovani* growth in murine infection, suggesting cytokine combinations may enable significant dose reduction of toxic drugs (Kumar et al., 2018; Kumar et al., 2019).

### 6.2 TLR agonist approaches

Toll-like receptor (TLR) agonists represent a more targeted immunomodulatory strategy, activating innate immune pathways to restore macrophage function and drive Th1 polarization. The combination of liposomal CpG oligodeoxynucleotide 2006 (a TLR9 agonist) with miltefosine induced strong cell-mediated immunity and reduced splenic parasite burden in experimental VL, outperforming miltefosine monotherapy. CpG sequences activate TLR9 in macrophages and plasmacytoid dendritic cells, driving IL-12 production and Th1 differentiation (Shivahare et al., 2014; Coler et al., 2007).

TLR4 agonists (including MPL-SE, a monophosphoryl lipid A derivative) have been used as adjuvants in multiple vaccine formulations (LEISH-F3 + GLA-SE, Leish-111f + MPL-SE), improving Th1 immunogenicity (Coler et al., 2007; Duthie et al., 2016). TLR5 agonists (flagellin) and TLR7/8 agonists have demonstrated antileishmanial and macrophage-activating properties in experimental models (Sharma et al., 2020; Thakur et al., 2025). The dual anti-inflammatory and immunostimulatory properties of galactosylated flavonoids, which activate TLR pathways while modulating regulatory responses, have also been reported (Pradhan et al., 2022). Notably, TLR9 signaling is critical for the protective efficacy of intranasal vaccination with whole Leishmania antigens: TLR9-deficient mice completely lose intranasal vaccine-mediated protection, suggesting that naturally occurring TLR9 ligands within Leishmania DNA are key to adjuvant-free vaccine formulations (Pratti et al., 2019).

### 6.3 Therapeutic and prophylactic vaccines

No licensed human vaccine for any form of leishmaniasis exists, representing one of the most significant failures in NTD vaccine development (Noazin et al., 2008; Gillespie et al., 2016; Palatnik-de-Sousa and Nico, 2020). Historically, leishmanization—deliberate infection with live *L. major*—provided solid protection in the Middle East and Central Asia but was abandoned due to unacceptable risk of severe lesions and HIV-era concerns about live pathogen use (Nadim et al., 2014). First-generation vaccines using killed whole parasites with BCG adjuvant demonstrated 76–98% cure rates in CL clinical trials in Brazil and Venezuela, establishing proof-of-concept for therapeutic vaccination (Mayrink et al., 2006; Roatt et al., 2014; Noazin et al., 2008).

Second-generation recombinant protein vaccines have dominated the preclinical pipeline. The LEISH-F3 + GLA-SE construct (fused polyprotein of TSA, LmSTI1, and LeIF with TLR4 adjuvant) achieved strong Th1 responses in Phase I clinical trials (Coler et al., 2007; Duthie et al., 2016). Chimeric protein vaccines combining multiple Leishmania antigens with saponin-based or TLR4 adjuvants have demonstrated parasite clearance in murine and hamster models, with superior outcomes in combination with amphotericin B or miltefosine compared to drug alone (Vale et al., 2023; Jesus et al., 2025; Mutiso et al., 2010). DNA vaccines using CRISPR-optimized attenuated Leishmania strains represent a third-generation approach with promising pre-clinical outcomes (Zhang et al., 2020; Ayala et al., 2024).

A critical observation across vaccine studies is the superiority of intranasal over parenteral delivery routes for inducing protective Th1 responses in Leishmania models. In BALB/c mice, intranasal administration of whole *L. amazonensis* antigens (LaAg) confers significant protection against CL, with reduced lesion size and parasite burden compared to subcutaneous or intramuscular delivery of the same antigen (Pratti et al., 2016; Pratti et al., 2019). In Syrian golden hamsters, intranasal LaAg attenuates *L. braziliensis* infection while intramuscular vaccination fails to protect (da Silva-Couto et al., 2015). For VL, intranasal immunization with *L. donovani* antigens outperforms intradermal delivery in parasite clearance across liver, spleen, and bone marrow (de Matos Guedes et al., 2014; da Silva-Couto et al., 2015; Helou et al., 2021).

An underappreciated dimension of vaccine-mediated protection in VL and PKDL is the CD8 + cytotoxic T lymphocyte (CTL) response. As described in Section 4.1, CD8 + CTLs mediate killing of infected macrophages via perforin/granzyme pathways and amplify IFN-γ production, particularly in visceral leishmaniasis (VL). Particulate vaccine delivery platforms — including Qβ VLPs, nanoparticles, and liposome-formulated antigens — are particularly effective at engaging the cross-presentation pathway in dendritic cells, directing antigen onto MHC class I complexes to prime CD8 + CTL responses alongside CD4 + Th1 activation (Lycke, 2012). DNA vaccines, by directly transfecting host antigen-presenting cells, naturally access the endogenous MHC class I presentation pathway and are among the most effective CTL-priming platforms in preclinical models. For VL, restoring CD8 + CTL function is mechanistically relevant for clearing infected macrophages in the spleen and bone marrow where extracellular antibody-mediated effectors have limited access. In PKDL, where dermal parasite persistence is associated with dysfunctional exhausted CD8 + T-cell infiltration, therapeutic vaccination aimed at CTL reinvigoration — potentially combined with checkpoint blockade — represents a rational and underexplored immunotherapy strategy.

### 6.4 *α*-Gal epitope targeting as an emerging immunotherapy

A mechanistically distinct immunotherapy approach leverages the α-Gal epitope (galactose-α-1,3-galactose-β-1,4-N-acetylglucosamine), a carbohydrate structure present on Leishmania surface glycoconjugates but absent in humans and Old World primates due to evolutionary inactivation of the α1,3-galactosyltransferase gene (Galili et al., 1985; Galili, 2021). Humans naturally harbor high titers of anti-α-Gal antibodies (constituting 1–3% of circulating IgG), generated through continuous exposure to α-Gal-expressing gut bacteria (Galili, 2020; Almeida et al., 1991). These pre-existing antibodies can be harnessed to drive antibody-dependent cellular cytotoxicity (ADCC) and complement-dependent cytotoxicity (CDC) against α-Gal-expressing Leishmania (Galili, 2020; Subramaniam et al., 2018; Moura et al., 2017). Complement-mediated lysis of parasites expressing α-Gal has been demonstrated experimentally, with anti-α-Gal antibodies engaging the classical complement pathway to achieve parasite killing (Almeida et al., 1991; Galili, 2015).

Protection was achieved without additional adjuvants, an observation attributed to the engagement of pre-existing anti-α-Gal antibodies that are present in high titers in humans and α1,3-galactosyltransferase knockout mice alike (Moura et al., 2017; Galili, 2021). The physical properties of Qβ VLPs—approximately 25 nm in diameter, high structural stability, and efficient uptake by mucosal M cells and dendritic cells in mucosa-associated lymphoid tissues (MALT)—have been noted as favorable attributes for intranasal administration compared to larger VLP platforms such as HBsAg and HPV L1 (Table 3). The documented co-expression of α-Gal on Leishmania surface glycoproteins and glycolipids, confirmed by reverse immunoglycomics on *L. major* and *L. donovani*, tentatively supports the Leishmania surface as a candidate target for anti-α-Gal antibody-mediated effector mechanisms (Montoya et al., 2021; Subramaniam et al., 2018).

**Table 3:** Comparison of selected VLP platforms for α-Gal display and mucosal delivery.

Platform	Size (nm)	Mucosal stability	Immune bias	Mucosal uptake	Key notes
Qβ bacteriophage	~25	High	Strong Th1	Excellent	Moura et al., 2017; efficient APC uptake
HBsAg	22–42	Moderate	Variable	Good	General VLP literature
CPMV (plant-based)	~30	Moderate	Moderate	Moderate	General VLP literature
HPV L1	50–60	High	Strong humoral	Moderate	General VLP literature

Qβ bacteriophage VLPs offer advantages for intranasal delivery based on small size, high mucosal stability, and Th1-biased immunogenicity. Data from Moura et al. (2017) and Galili (2020, 2021). Platforms included are restricted to those with published data in Leishmania antigen delivery or alpha-Gal epitope display contexts. APC, antigen-presenting cell (Moura et al., 2017).

It is important to note that Moura et al. (2017) employed alpha-1,3-galactosyltransferase knockout (KO) mice as the immunization model. While KO mice, like humans, lack endogenous alpha-Gal expression and thus mount anti-alpha-Gal antibody responses upon immunization, the study assessed antibody induction and *L. major* susceptibility in a cutaneous model — not a therapeutic or prophylactic intervention against an established visceral *L. donovani* infection. To our knowledge, alpha-Gal VLP immunotherapy has not been tested in any *in vivo* visceral leishmaniasis model with established infection. This gap constitutes the most critical preclinical milestone before the platform can be considered a serious candidate for VL immunotherapy.

Key gaps remain before α-Gal VLP immunotherapy can progress toward clinical application. Efficacy in visceral models against established *L. donovani* infection—where parasites persist in reticuloendothelial organs—has not been tested. Optimal VLP formulation parameters for intranasal delivery (α-Gal epitope density, particle size, mucosal adjuvant compatibility) require systematic optimization (Galili, 2015; Galili, 2020). Human anti-α-Gal antibody variability—driven by microbiome composition differences between endemic and non-endemic populations—introduces uncertainty about the immunogenicity of α-Gal-based platforms in the populations most affected by VL. These gaps define a critical preclinical research agenda for this emerging platform.

## 7 Immunochemotherapy approaches

### 7.1 Rationale for combining drug and immune therapy

Before reviewing this evidence, a critical caveat must frame the entire immunochemotherapy section: to date, no immunotherapy or immunochemotherapy approach — beyond IFN-*γ* combined with pentavalent antimonials in early clinical trials — has demonstrated efficacy in a Phase II/III randomized controlled trial for any form of leishmaniasis. All immunochemotherapy sterile cure data discussed in Section 7.2 derive from preclinical rodent, hamster, and canine models. This distinction between preclinical proof-of-concept and clinically validated therapy is fundamental, and we return to it in Section 8.

The limitations of chemotherapy alone—incomplete immune restoration, relapse, and resistance—and the limitations of immunotherapy alone—insufficient parasite reduction without active drug killing—create a compelling rationale for immunochemotherapy combinations (Roatt et al., 2014; Yadagiri et al., 2023; Badaro et al., 1986). In this framework, the drug component (miltefosine, amphotericin B, or antimonials) reduces the acute parasite burden, alleviating the immunosuppressive environment driven by high parasite loads, while the immunological component (vaccine, cytokine, TLR agonist, or novel biological) restores and sustains protective Th1 immunity to prevent relapse and establish long-term protection.

The synergistic potential is mechanistically grounded. High parasite loads in VL directly suppress T-cell function via IL-10, IL-27, and PD-1/PD-L1 upregulation; partial parasite clearance by drug treatment creates an immunological window during which vaccines or immunomodulators can most effectively prime Th1 responses (Faleiro et al., 2014; Dayakar et al., 2019). Conversely, vaccine-primed Th1 immunity enhances macrophage killing capacity, potentially enabling dose reduction of toxic drugs while maintaining or improving cure rates (Roatt et al., 2014; Yadagiri et al., 2023; Haldar et al., 2011). The result is a mutually amplifying combination with theoretical advantages across parasite clearance, relapse prevention, dose reduction, and resistance mitigation (Roatt et al., 2014; Yadagiri et al., 2023).

### 7.2 Evidence base for Immunochemotherapy

The strongest evidence for immunochemotherapy efficacy comes from heterologous vaccine therapy combined with miltefosine in the Syrian golden hamster model of VL, which most faithfully recapitulates human hepato-splenomegaly, progressive weight loss, and fatal outcome (Carvalho et al., 2022; Goto et al., 2007). Carvalho et al. (2022) demonstrated that heterologous vaccine therapy combined with half-course miltefosine achieved sterile cure—complete parasite clearance—in *L. donovani*-infected hamsters, significantly outperforming either component alone. The combination activated proinflammatory responses, restored Th1 cytokine dominance, and controlled splenic parasitism with a miltefosine dose that was insufficient for cure on its own, demonstrating genuine dose-sparing (Carvalho et al., 2022).

Chimeric recombinant protein vaccines combined with standard drug therapy have shown superior outcomes in multiple preclinical models. Jesus et al. (2025) demonstrated that an immunotherapeutic regimen combining a recombinant chimeric protein, MPL adjuvant, and miltefosine achieved superior parasite clearance and immune restoration in murine VL compared to drug alone. Vale et al. (2023) similarly showed that an immunogenic chimeric protein combined with amphotericin B outperformed monotherapy in murine VL (Vale et al., 2023). In canine VL models—clinically relevant as dogs are the primary reservoir of *L. infantum*—immunochemotherapy with recombinant Leish-110f + MPL-SE vaccine and meglumine antimoniate demonstrated superior outcomes compared to drug alone (Miret et al., 2008; Gonçalves et al., 2019).

Earlier immunotherapy studies established that IFN-*γ* combined with antimonials substantially improved cure rates in both murine and human VL clinical trials, providing the foundational clinical proof-of-concept for immune augmentation during antileishmanial chemotherapy (Sundar et al., 2004; Murray, 1997). TLR9 agonist (liposomal CpG 2006) combined with miltefosine induced stronger cell-mediated immunity and greater splenic parasite reduction in experimental VL than miltefosine alone, consistent with the synergistic Th1-amplifying mechanism expected from TLR9 activation (Shivahare et al., 2014). Immunochemotherapy for PKDL has also been explored clinically, with combination regimens achieving resolution of otherwise recalcitrant dermal lesions (Musa et al., 2008; Zijlstra, 2016).

Regarding the clinical translation landscape: IFN-γ combined with pentavalent antimonials achieved 88–100% cure rates in clinical trials in India and East Africa versus antimonials alone (Murray, 1997), establishing the principle of immune augmentation during antileishmanial chemotherapy in humans and providing the only Phase III-level immunotherapy efficacy data currently available. The LEISH-F3 + GLA-SE recombinant protein vaccine completed Phase I/IIa trials demonstrating acceptable safety and Th1-polarized immunogenicity in healthy adults and at-risk volunteers, but these early-phase studies were not designed or powered to assess parasite clearance efficacy endpoints (Duthie et al., 2016; Coler et al., 2007). No contemporary immunochemotherapy combination — pairing a vaccine or novel biological platform with miltefosine, liposomal amphotericin B, or paromomycin — has entered Phase II/III evaluation with a VL efficacy endpoint. This preclinical-to-clinical translation gap is driven in part by the absence of validated immunological correlates of protection in humans, which are needed to enable rational endpoint selection and patient stratification for clinical trials.

### 7.3 Mucosal Immunochemotherapy

Despite the documented advantages of mucosal vaccination for Th1 induction in Leishmania models, immunochemotherapy combining mucosal delivery routes with standard antileishmanial drugs remains essentially unexplored. The available evidence from mucosal vaccine studies is instructive: intranasal vaccines achieve protection in models where parenteral vaccines fail, and this protection is associated with superior Th1 cytokine production, SIgA induction, and tissue-resident memory T cell establishment at sites relevant to Leishmania persistence (Helou et al., 2021; de Matos Guedes et al., 2014; da Silva-Couto et al., 2015; Holmgren and Czerkinsky, 2005; Lycke, 2012).

The pharmacological profile of miltefosine—oral bioavailability, long terminal half-life (~150 h), and documented Th1-skewing immunomodulatory effects including upregulation of MHC II and costimulatory molecules on macrophages—has been discussed in the context of potential compatibility with mucosal immunotherapy approaches (Shivahare et al., 2014; Dorlo et al., 2012; Sundar and Olliaro, 2007). Whether mucosal SIgA induction and systemic Th1 priming achieved by intranasal vaccination would be additive or synergistic with miltefosine’s immunomodulatory properties has not been examined experimentally, and the safety profile of such combinations, including local mucosal tolerance and drug absorption kinetics, remains uncharacterized.

## 8 Knowledge gaps and future directions

Despite significant advances in understanding *Leishmania* pathogenesis, host immunity, and the preclinical evidence base for immunotherapy and immunochemotherapy, several critical gaps prevent translation to clinical practice.

First, no immunotherapy or immunochemotherapy approach has yet demonstrated efficacy in a Phase II/III clinical trial for VL, and the field lacks validated immunological correlates of protection in humans that would guide rational vaccine design and endpoint selection for clinical trials (Gillespie et al., 2016; Palatnik-de-Sousa and Nico, 2020). Surrogate markers of protective immunity (IFN-γ: IL-10 ratios, TRM persistence, IgG avidity indices) require prospective validation in endemic cohort studies.

Second, the optimal timing, sequence, and dosing of immunotherapy relative to chemotherapy remain incompletely defined (Yadagiri et al., 2023; Roatt et al., 2014). The immunological window created by drug-induced parasite reduction—within which immunotherapy may be most effective—has not been systematically characterized. The question of whether vaccination should precede infection (prophylactic), occur during active disease (therapeutic), or begin at the point of drug treatment initiation (concurrent) has different answers for different platforms and endpoints.

Third, human anti-α-Gal antibody variability—driven by microbiome composition differences between endemic and non-endemic populations—introduces uncertainty about the immunogenicity of α-Gal-based platforms in the populations most affected by VL (Galili, 2020; Galili, 2021; Galili et al., 1985). Baseline anti-α-Gal titer assessment in VL-endemic populations in South Asia and East Africa is needed to predict therapeutic window and identify responder subgroups.

Fourth, the role of the sandfly vector in shaping the initial immune response at the bite site is increasingly appreciated. Salivary proteins from Phlebotomus and Lutzomyia species modulate early innate and adaptive immunity, and vector-derived antigens are being incorporated into next-generation VL vaccines (Pandey and Prajapati, 2020). Integrating vector-targeted components into combination strategies may enhance protection at the point of parasite entry.

Fifth, the neglected animal reservoir—particularly dogs in zoonotic *L. infantum* transmission cycles—represents both a translational challenge and an opportunity. Effective canine VL vaccines could interrupt transmission to humans without requiring human trials; three licensed canine VL vaccines exist in Brazil (Leish-Tec®, Leishmune®, and CaniLeish®) and provide an established regulatory pathway and real-world immunogenicity and safety database (Gonçalves et al., 2019; Dube et al., 1998). Immunochemotherapy protocols validated in canine models would provide high translational confidence for human trials.

Finally, the drug development pipeline for leishmaniasis remains critically underfunded and thin (Chatelain and Ioset, 2011). Fexinidazole, a nitroimidazole originally developed for sleeping sickness, has shown activity against VL in early clinical evaluation and represents a potential new oral agent (Drugs for Neglected Diseases Initiative, 2023). Drug repurposing approaches and structure–activity relationship studies of heterocyclic compounds continue to yield antileishmanial lead candidates (Roussaki et al., 2012; Singh et al., 2012). Combination of novel compounds with immunotherapy represents the most plausible path to durable, curative, and resistance-resistant treatment in the near term (Bhusal et al., 2025; Zhang et al., 2025; Chatelain and Ioset, 2011).

## 9 Conclusion

Leishmaniasis remains a major global health challenge for which the current chemotherapeutic arsenal is insufficient—limited by toxicity, resistance, high cost, parenteral delivery requirements, and universal failure to restore durable protective immunity (Croft et al., 2006; Ponte-Sucre et al., 2017; Murray et al., 2005). The disease is fundamentally an immunological failure: Leishmania succeeds as a parasite not because it is unkillable, but because it has evolved a sophisticated capacity to subvert the very macrophages that should eliminate it (Olivier et al., 2005; Tibúrcio et al., 2019).

The immunotherapy and immunochemotherapy evidence reviewed here—from cytokine and TLR agonist combinations to therapeutic vaccines and novel platforms such as α-Gal VLPs—collectively indicates that immune restoration can amplify chemotherapy efficacy, enable dose reduction, prevent relapse, and in some preclinical models achieve sterile cure outcomes not attainable by drugs alone (Carvalho et al., 2022; Jesus et al., 2025; Vale et al., 2023; Roatt et al., 2014). Mucosal delivery routes, particularly intranasal administration, have demonstrated advantages for Th1 polarization, SIgA induction, and tissue-resident memory T cell establishment in animal models (Lavelle and Ward, 2021; Holmgren and Czerkinsky, 2005; Lycke, 2012; Corthésy, 2013), though their translation to clinical immunochemotherapy combinations remains largely unexplored.

Several areas warrant further investigation: validation of immunological correlates of protection in human cohorts; characterization of the immunological window created by antileishmanial chemotherapy within which immune priming is most effective; safety and efficacy evaluation of mucosal immunochemotherapy in established animal models; and assessment of anti-α-Gal antibody titers and variability in VL-endemic populations to establish the potential therapeutic window for α-Gal-based platforms (Galili, 2020; Galili et al., 1985; Pandey and Prajapati, 2020).

Progress across these fronts will depend on sustained investment in neglected tropical disease research (Chatelain and Ioset, 2011) and coordinated translation from preclinical models—particularly the Syrian hamster, canine, and non-human primate models that most faithfully recapitulate human disease (Gonçalves et al., 2019; Dube et al., 1998)—to clinical evaluation. The evidence reviewed here identifies combination immunochemotherapy as the most mechanistically coherent path toward durable, curative therapy for leishmaniasis, though substantial experimental and clinical work remains before any specific combination approach can be recommended for practice.

Leishmaniasis persists as a global health burden that reflects an immunological failure as much as a pharmacological one: Leishmania succeeds not because it is unkillable, but because it has evolved a sophisticated capacity to subvert the macrophages that should eliminate it. The current chemotherapeutic arsenal remains insufficient — limited by toxicity, resistance, high cost, parenteral delivery requirements, and failure to restore durable protective immunity — and monotherapy alone is unlikely to change that calculus.

The evidence reviewed here — from cytokine-based and TLR agonist immunotherapy to therapeutic vaccines and novel platforms such as alpha-Gal VLPs — collectively demonstrates that immune restoration can amplify chemotherapy efficacy, enable dose reduction, prevent relapse, and in select preclinical models achieve sterile cure outcomes not attainable by drugs alone. The Syrian golden hamster data from Carvalho et al. (2022), in which heterologous vaccine therapy combined with a half-course of miltefosine achieved sterile cure, remains the strongest single preclinical proof-of-concept in the field. Mucosal delivery routes, particularly intranasal administration, offer documented advantages for Th1 polarization, SIgA induction, and tissue-resident memory T-cell establishment in animal models, though their application requires resolution of formulation, safety, and translatability challenges before clinical advancement.

Our assessment of translational priority: miltefosine’s immunomodulatory profile — Th1 polarization, MHC II upregulation, and restoration of T-cell proliferative capacity in anergic VL patients — makes it the most rationally positioned drug partner for immunotherapy combinations and the priority backbone for near-term clinical immunochemotherapy trials. Among vaccine platforms, chimeric recombinant protein vaccines with TLR4 adjuvants have the best-characterized Phase I safety and immunogenicity profiles and are closest to clinical combination testing. DNA vaccine platforms and VLP-based systems are mechanistically well-suited for CTL priming and should be advanced to Syrian golden hamster combination studies. The alpha-Gal VLP platform has a compelling mechanistic rationale and a unique target — exploiting the largest natural antibody pool in humans against a confirmed parasite surface glycan absent in humans — but remains the most distal from clinical application and requires VL challenge model data before its translational potential can be meaningfully assessed.

Our prioritized research agenda: (1) validate immunological correlates of protection — IFN-gamma: IL-10 ratios, tissue-resident memory T-cell persistence, IgG avidity indices — in prospective human cohort studies in VL-endemic regions; (2) characterize the immunological window created by antileishmanial chemotherapy within which immune priming is most effective; (3) evaluate alpha-Gal VLP immunotherapy in Syrian golden hamster *L. donovani* models with and without miltefosine; (4) assess baseline anti-alpha-Gal antibody titers in VL-endemic populations to define the immunogenicity window; (5) advance the most promising preclinical immunochemotherapy combination to Phase I/II clinical evaluation. Progress across these fronts will depend on sustained investment in neglected tropical disease research and coordinated translation from preclinical models to clinical evaluation. The evidence reviewed here identifies combination immunochemotherapy as the most mechanistically coherent path toward durable, curative therapy for leishmaniasis, and the field now has the preclinical foundation to move decisively toward that goal.
